# Aroma Profile of Worts and Contents of Selected Mineral Elements in Brewing with Malted and Unmalted Hemp Seeds

**DOI:** 10.3390/molecules31050809

**Published:** 2026-02-28

**Authors:** Robert Duliński, Marek Zdaniewicz, Jana Lakatošová, Adam Florkiewicz, Janusz Gołaszewski, Bożena Bukowska

**Affiliations:** 1 Department of Biotechnology and General Food Technology, Faculty of Food Technology, University of Agriculture in Krakow, Balicka Street 122, 30-149 Krakow, Poland; 2 Department of Fermentation Technology and Microbiology, Faculty of Food Technology, University of Agriculture in Krakow, Balicka Street 122, 30-149 Krakow, Poland; m.zdaniewicz@urk.edu.pl; 3 AgroBioTech Research Centre, Slovak University of Agriculture in Nitra, Tr. A. Hlinku 2, 949 76 Nitra, Slovakia; jana.lakatosova@uniag.sk; 4 Department of Food Analysis and Quality Assessment, Faculty of Food Technology, University of Agriculture in Krakow, 122 Balicka St., 30-149 Krakow, Poland; adam.florkiewicz@urk.edu.pl; 5 Department of Food Technology and Security, Faculty of Health Sciences, University of Lomża, 14 Akademicka Str., 18-400 Łomża, Poland; jgolaszewski@al.edu.pl; 6 Department of Biophysics of Environmental Pollution, Faculty of Biology and Environmental Protection, University of Lodz, Pomorska Street 141/143, 90-236 Lodz, Poland; bozena.bukowska@biol.uni.lodz.pl

**Keywords:** volatile organic compounds, wort, hemp seeds, GC-MS, brewing, 2,3-butanediol, aroma profile, PCA

## Abstract

The growing interest in functional beer production has led to the exploration of unconventional raw materials, such as hemp (*Cannabis sativa* L.), for brewing applications. This study aimed to evaluate the volatile organic compound (VOC) profile and the macro- and microelement composition of barley wort enriched with varying proportions (10% and 30%) of malted and unmalted hemp seeds, using solid-phase microextraction followed by gas chromatography–mass spectrometry (SPME–GC–MS) and atomic absorption spectrometry (AAS). A total of 64 VOCs were identified across four wort variants: control (barley malt only), 10% malted hemp, 30% malted hemp, and 30% unmalted hemp. The aroma profile was significantly influenced by compounds such as 2,3-butanediol, 1-hexanol, 3-methyl-1-butanol, 3-hydroxy-2-butanone, hexanoic acid, and 4-vinylguaiacol (*p* < 0.001). Principal component analysis (PCA) revealed clear separation between wort types based on the relative abundance of alcohols, acids, ketones, and phenols, indicating a progressive shift from sweet/malty toward acidic, green, and herbal aroma notes as hemp addition increased. Notably, unmalted hemp seeds resulted in a pronounced dominance of hexanoic acid, which may contribute to earthy and rancid sensory attributes. The evaluation of selected mineral elements showed that the key macroelements differentiating the worts were potassium, magnesium, phosphorus, and calcium, while among the microelements the distinguishing elements were manganese, iron, and sodium. These findings demonstrate the strong modulating effect of aromatic hemp-derived materials on the aroma composition and selected mineral content of brewing worts, supporting their targeted use in novel beer formulations.

## 1. Introduction

In recent years, the brewing industry has witnessed a growing interest in the incorporation of unconventional ingredients to enhance both the nutritional and sensory attributes of beer [1,2]. Among such novel additions, hemp (*Cannabis sativa* L.) has gained attention due to its rich composition of bioactive compounds, including cannabinoids, polyphenols, and aroma-active molecules such as terpenes and alcohols [3,4,5]. *Cannabis sativa* L. exhibits a diverse profile of volatile compounds depending on the cultivation region, and its volatile composition effectively reflects the geographical origin of the raw material. Song et al. (2022) [6] conducted a comprehensive analysis of the chemical composition, including volatile compounds, in hemp seeds originating from Dongbei, Guangxi, Inner Mongolia, and Shanxi in China. More than 300 volatile compounds were detected, and among 110 volatiles with odour activity values (OAVs), 35 key contributors to the characteristic aroma were ultimately identified. These included 14 aldehydes, 2 ketones, 4 alcohols, 2 esters, 1 acid, 3 aromatic compounds, 5 terpenes, and 4 other compounds [6]. Kamiński et al. highlight the rich and complex sensory profile of *Cannabis sativa* L., which results from a wide spectrum of volatile compounds—such as terpenes, aldehydes, ketones, esters, and sulfur-containing molecules—as well as non-volatile taste-active substances, including flavonoids and phenolic compounds [4]. These compounds collectively define the plant’s characteristic aroma and flavour [4]. Terpenes derived from cannabis—myrcene, limonene, linalool, and caryophyllene—offer a wide range of aromatic qualities, ranging from earthy and spicy to tropical/fruity and subtly floral [7,8].

The application of hemp in brewing enables the development of new, sophisticated sensory profiles in beer. However, strategic dosing and careful integration with the existing aroma profile of the beer are crucial, requiring the use of precise analytical and sensory methods [9].

Although the application of hemp derivatives—such as hemp seed oil or CBD extracts—has been explored primarily in the context of functional beverages [10,11,12], relatively little is known about the impact of whole hemp seeds, whether malted or unmalted, on the aroma profile of brewing worts.

Volatile organic compounds (VOCs) are key determinants of beer flavour and aroma, influencing consumer preference and product differentiation. Their composition in wort is shaped by both the type and proportion of raw materials used, as well as by enzymatic and thermal reactions occurring during mashing and boiling. Previous studies have demonstrated that the addition of non-barley adjuncts can significantly alter the volatile profile, potentially affecting the sensory characteristics of the final product. However, comprehensive data on VOCs in hemp-enriched wort remain scarce [13,14].

*Cannabis sativa* L. is also a valuable source of essential mineral elements. Numerous studies have shown that both seeds and vegetative parts of the plant contain a diverse range of macro- and microelements crucial for human nutrition and plant metabolism. Among the most abundant macronutrients found in *Cannabis sativa* seeds are potassium (K), phosphorus (P), magnesium (Mg), and calcium (Ca), while trace elements such as iron (Fe), zinc (Zn), manganese (Mn), copper (Cu), and selenium (Se) have also been reported in significant concentrations [15,16]. Hemp seeds are a rich source of potassium (approximately 690–900 mg per 100 g), phosphorus (410–510 mg/100 g), and magnesium (around 420–460 mg/100 g), making them nutritionally valuable in the human diet [17]. These minerals are essential for enzymatic regulation, energy metabolism, and antioxidant defense mechanisms.

Beer contains a variety of essential ions, primarily cations—calcium, magnesium, sodium, and potassium—and anions such as sulfate, nitrate, phosphate, chloride, and silicate, all of which play important roles in its chemical and sensory properties. In contrast, iron, copper, zinc, and manganese generally considered less influential in this regard [18]. In brewing, the mineral composition of raw materials crucially shapes multiple stages of the process—from mashing through fermentation and final flavour development. Ions are essential not only for proper fermentation and the development of beneficial microorganisms but also influence the taste of beer through their role as non-volatile, flavour-active compounds [18,19]. Calcium, for example, supports protein coagulation, improves beer clarity, and is instrumental in lowering mash pH into the optimal 5.2–5.6 range, which enhances the efficiency of starch-converting enzymes such as α and β amylase. Taylor & Daiber [20] demonstrated in sorghum mashing that approximately 200 ppm of calcium significantly increases reducing sugar yield by maintaining α amylase activity at lower pH levels. Magnesium (Mg^2+^) is a cofactor for more than 300 enzymes [21,22], although used in excess it can impart a bitter aftertaste to beer [23]. Another important metal, sodium (Na^+^), at moderate concentrations, enhances mouthfeel and brings out the malt’s sweetness [24]. Potassium (K^+^) plays an important role as a cofactor in yeast growth and in supporting carbohydrate metabolism; however, in excessive concentrations—above ~500 mg/L—it can impart a distinct salty flavour to beer and suppress mash enzymes [24]. Trace elements such as zinc, manganese, and phosphate are equally critical, as they serve as cofactors in enzymatic reactions within yeast, promoting robust fermentation, higher alcohol conversion, and balanced ester formation [25,26].

Even minor deviations in mineral profiles can influence mash efficiency [27], yeast flocculation [23], and the sensory experience—ranging from mouthfeel to bitterness and residual sweetness [28]. In the context of brewing, the mineral content of raw materials can significantly influence mash pH, enzyme activity, yeast metabolism, and the final flavour profile. The introduction of *Cannabis sativa* L. seeds or derivatives into the brewing process may therefore not only contribute bioactive compounds but also modify the mineral composition of wort and beer, potentially impacting both fermentation dynamics and sensory attributes.

Accordingly, this study tested the hypothesis that both the level of hemp seed addition and its technological state (malted versus unmalted) act as key modulating factors influencing wort chemical composition and volatile aroma profiles, using a comparative, semi-quantitative approach. Using headspace SPME-GC-MS and atomic absorption spectrometry (AAS), we evaluated the qualitative and quantitative differences in aroma compound profiles and macro- and microelements across four wort formulations. The impact of hemp addition on the relative abundance of key compound classes such as alcohols, acids, ketones, and phenols was assessed, and specific VOCs were identified as chemical markers associated with the presence of hemp-derived material.

These results provide valuable insight into the modulation of beer aroma precursors and macro- and microelements through the use of hemp as a brewing adjunct. This study contributes to the growing body of research on sustainable and health-oriented innovations in brewing, highlighting *Cannabis sativa* L. as a valuable raw material for developing novel beer styles with enhanced chemical and sensory complexity.

## 2. Materials and Methods

### 2.1. Materials

Hemp seeds of the Henola variety were used. The seeds were cultivated in Poland and supplied for research by Hemp Farm Poland (Krakow, Poland). Barley grains malted by Ireks (Kulmbach, Germany) were used as a reference.

### 2.2. Malting Procedure

The process of malting hemp seeds was carried out under laboratory conditions at the Department of Fermentation Technology and Microbiology, University of Agriculture in Krakow. The process consisted of three main stages: soaking the grain, germination, and drying. The total soaking time was six hours and comprised nine water and nine air cycles. For this purpose, 100 g of seeds were soaked in 500 mL of water at 19 °C for 30 min, after which the water was removed, and the seeds were left out to rest for five minutes while being continuously shaken. Upon completion of the required number of soaking cycles, the samples were weighed and transferred for germination. During germination, the soaked seeds were maintained at a temperature of 20 °C. Every 24 h, their surface was sprinkled, and the entire batch was mixed. The germinated seeds were dried at 50 °C for five hours to reach the desired moisture level (below 4% *m*/*m*).

### 2.3. Mashing

Barley malt (IREKS, Kulmbach, Germany) with the addition of the hemp seeds (whole untreated seeds were used; no dehulling or oil extraction was applied) brews were prepared following a method similar to that used for barley malt (EBC 4.5.1). For this purpose, 50 g of malt, milled in a laboratory grinder, was weighed into tarred mash containers, which were then placed in a water-heated apparatus at 45 °C. Agitators were fitted, and the “Congress programme” was selected. Next, 200 mL of distilled water at 45 °C was added in portions to the containers. The apparatus was held at 45 °C for 30 min. The temperature was then raised at a rate of 1 °C per minute until it reached 70 °C, with constant stirring of samples. When the temperature reached 70 °C, 100 mL of distilled water warmed to the same temperature was added to the cups, and the set temperature was maintained for one hour. Afterwards, the containers were cooled to 20 °C, topped up with distilled water to a total mass of 450.0 g, and filtered through a paper filter. To ensure high clarity, the first portions of the filtrate were recirculated.

### 2.4. VOC Determination

The sample preparation was carried out according to Starzyńska-Janiszewska [29] with a modification. A wort sample (2.0 mL) with approximately 0.8 g NaCl in a 20 mL headspace vial was extracted using a headspace SPME fibre with DVB/CAR/PDMS (50/30 µm, Supelco, Bellefonte, PA, USA) via an autosampler CTC120 (CTC Analytics AG, Zwingen, Switzerland) at 40 °C for 30 min. Analytes trapped in the fibre were desorbed for one minute in the injector of a gas chromatography–mass spectrometry (GC-MS) system (GC 7890B coupled with MSD 5977A; Agilent Technologies Inc., Waldbronn, Germany) and injected in split mode (20:1). GC-MS conditions were set according to Tomáška et al. [30] with a modification. A HP-5ms column (30 m × 0.25 mm × 0.25 µm; Agilent Technologies Inc.) was used. The oven temperature programme started at 40 °C, was held for one minute, then increased at a rate of 3 °C/min to 230 °C, where it was held for five minutes. Helium was used as the carrier gas at a constant flow rate of 1.2 mL/min. The mass detector parameters were as follows: ionisation energy of the filament, 70 eV; transfer line temperature, 250 °C; MS source temperature, 230 °C; quadrupole temperature, 150 °C. The mass spectrometer operated under electron impact (EI) mode in a full-scan mode at *m*/*z* 40–450 with a scan rate of 1.8 scans/s. Each sample was measured in triplicate.

Compound identification was carried out by comparing mass spectra (over 80% match) with those in the commercial database NIST 2017 library (National Institute of Standards and Technology, Gaithersburg, MD, USA) and Wiley library, as well as by comparing the occurrence of compounds in wort against the literature [31,32,33,34]. The relative percentage (%) of each volatile compound was calculated by dividing the individual peak area by the total area of all peaks. Peaks below 0.1% relative area was excluded from Table 1 and denoted “nd” (not detected); method LOD ~0.01–0.05% relative area for most VOCs.

The volatile aroma profile was determined exclusively in the produced wort samples, as the objective of the study was to evaluate the technological impact of hemp seed addition (malted and unmalted) on wort composition. The aroma profile of hemp seeds alone, prior to incorporation into the malt matrix, was not analysed.

### 2.5. Atomic Absorption Spectrometry (ASA)

The determination of mineral element content—specifically calcium, magnesium, potassium, and sodium—was carried out using the validated atomic absorption spectrometry method with flame atomisation FAAS (FAAS; Varian AA240FS, Varian Inc., Palo Alto, CA, USA) in accordance with the PN-EN 15505:2008 standard [35]. The determination of iron and zinc was carried out according to the PN-EN 14084:2004 standard [36]

Wet mineralisation was conducted using a microwave digestion method (MARSX-Press, CEM Corporation, Matthews, NC, USA) with nitric acid (Suprapur, Merck, Darmstadt, Germany; catalogue No. 1.00441). The process was conducted in 50 mL Teflon vessels, with the maximum temperature set to 200 °C.

Quantitative determination was based on external multi-point calibration. For each analytical series, fresh calibration curves were prepared to ensure analytical accuracy and to minimise instrumental drift. Depending on elemental response characteristics, linear or rational calibration models were applied. The obtained calibration curves showed excellent correlation coefficients (r = 0.9978–1.0000). The calibration ranges covered the expected concentration levels in wort samples, with upper limits of 20 mg/dm^3^ for Ca, K, and Na, and 4 mg/dm^3^ for Mg, Mn, Fe, and Zn. The use of stored curve libraries was intentionally avoided to ensure reproducibility and compliance with good analytical practice.

For the assessment of potassium and sodium content, a buffer solution was applied according to Schuhknecht and Schinkel (Merck, Darmstadt, Germany, catalogue No. 102037) at 2 mL per 50 mL of sample. For the assessment of calcium and magnesium content, a buffer solution was used according to Schinkel (Merck, Darmstadt, Germany, catalogue No. 1.16755) at 10 mL per 50 mL. As part of quality control, Certified Reference Material NCS ZC 73009 (China National Analysis Center for Iron and Steel, Beijing, China) was tested. Each sample was measured in triplicate.

### 2.6. Statistical Analysis

The nonparametric Kruskal–Wallis test was used to compare the aroma profiles of worts containing different percentages of malted and unmalted hemp seeds. Statistical analysis began with testing for normality of distribution using the Shapiro–Wilk test and for homogeneity of variance using Levene’s test, which showed that the ANOVA assumptions were not met for 68% of the VOCs. Given the small sample size (*n* = 3) and frequent violation of ANOVA assumptions, the non-parametric Kruskal–Wallis test was applied as a robust, distribution-free alternative despite its inherently limited statistical power under these conditions.

In the next stage of statistical analysis, principal component analysis (PCA) was performed based on the percentages of all 64 identified VOCs across four wort variants: control (barley malt), 10% hemp malt, 30% hemp malt, and 30% unmalted hemp seed. To visualise the separation of wort types and the directions of variability of selected chemical compounds (VOCs), a biplot of the first two principal components was generated.

For mineral composition data, normality and homogeneity of variance assumptions were met; therefore, one-way ANOVA followed by Tukey’s post hoc test was applied to identify significant differences between wort variants.

## 3. Results and Discussion

### 3.1. Aroma Profile of Worts

Comparative analysis of the chemical profiles of four wort types—control (barley malt), with the addition of 10% hemp malt, 30% hemp malt, and 30% unmalted hemp seed—showed that among the 64 identified VOCs, the dominant compounds with key influence on aroma were 2,3-butanediol, 3-methyl-1-butanol, 1-hexanol, 2-butanone-3-hydroxy, hexanoic acid, and 4-vinylguaiacol (Table 1). These chemical compounds significantly differentiated the aroma profiles of the worts (*p* < 0.001, Kruskal–Wallis test).

**Table 1:** Average concentration of volatile compounds in worts with malted and unmalted hemp seeds (% area).

No	Compound	Control Barley Malt		+10% Malted Hemp Seeds		+30% Malted Hemp Seeds		+30% Unmalted Hemp Seeds	
1	(Z,E) and (E,E)-3,5-octadien-2-one	nd		0.603	a	0.799	a	nd	
2	1,2-Benzenedicarboxylic acid, diethyl ester	nd		nd		0.173		nd	
3	1,3,5-Cycloheptatriene	nd		0.247		nd		nd	
4	1-Butanol, 3-methyl-	6.114	a	5.568	a	4.328	ab	1.754	b
5	1-Heptanol	0.334	b	0.487	ab	0.684	a	0.433	ab
6	1-Hexanol	2.509	b	5.762	a	10.556	a	5.120	ab
7	1-Hexanol, 2-ethyl-	0.190	a	0.273	a	0.200	a	nd	
8	1-Nonanol	0.230	a	0.163	a	nd		nd	
9	1-Octanol	0.427	a	0.582	a	0.625	a	nd	
10	1-Octen-3-ol	0.678	a	1.223	a	1.517	a	0.672	a
11	1-Pentanol	0.370	b	0.569	ab	1.073	a	0.378	ab
12	1-phenyl-ethanone	0.583	a	0.300	ab	0.170	b	0.475	ab
13	1-Propanol, 2-methyl-	0.295	a	0.250	b	nd		nd	
14	2(3H)-Furanone, 5-ethyldihydro-	nd		nd		nd		0.315	
15	2,3-Butanediol	22.257	a	22.261	a	3.504	b	1.206	b
16	2,4-Dimethylfuran	nd		1.767		nd		nd	
17	2,4-Di-tert-butylphenol	0.260	a	0.571	a	0.242	a	nd	a
18	2-Butanone, 3-hydroxy-	11.819	a	10.049	a	4.128	ab	1.763	b
19	2-Buten-1-one, 1-(2,6,6-trimethyl-1,3-cyclohexadien-1-yl)-	nd		nd		0.278		nd	
20	2-Buten-1-one, 1-(2,6,6-trimethyl-1,3-cyclohexadien-1-yl)-, (E)-	nd		nd		nd		0.370	
21	2-Heptanone	0.283	b	0.630	ab	0.714	a	0.787	a
22	2-Methoxy-4-vinylphenol	nd		nd		1.300		nd	
23	2-Nonanone	0.562	a	0.927	a	1.040	a	nd	
24	2-Octanone	nd		0.722		nd		nd	
25	3,5-Octadien-2-one	nd		nd		0.833	a	0.597	a
26	3-Cyclohexene-1-methanol, alpha,alpha,4-trimethyl-	nd		nd		0.240		nd	
27	3-Ethylcyclopentanone	nd		nd		0.270		nd	
28	3-Methylbutanoic Acid	nd		0.273		nd		nd	
29	3-Methylene-6-methyl-(hexahydro)benzofuran	nd		nd		0.273		nd	
30	4-vinyl-guaiacol	6.150	a	3.983	ab	1.257	b	0.853	b
31	4H-3,1-Benzoxazin-4-one, 2-ethoxy-	nd		nd		nd		1.240	
32	4-vinylphenol	nd		nd		0.665	a	0.917	a
33	Acetic acid	0.913	ab	0.225	b	1.571	ab	3.468	a
34	Benzaldehyde	nd		0.448	b	0.940	ab	1.383	a
35	Benzeneacetaldehyde	nd		0.433	a	0.331	a	0.301	a
36	Benzofuran, 2,3-dihydro-	2.228	a	1.023	b	1.103	ab	0.580	b
37	Butanoic acid, 2-methyl-	0.323	a	0.730	a	0.293	a	0.355	a
38	Butanoic acid, 3-methyl-	0.756	a	0.811	a	0.517	a	0.532	a
39	Cyclohexanol, 5-methyl-2-(1-methylethyl)-	nd		0.433	a	0.537	a	nd	
40	Dimethyl trisulfide	nd		0.260		nd		nd	
41	Ethyl-3-ethoxy-3-hydroxy-2-(2-hydroxybenzoyl)acrylate	0.647		nd		nd		nd	
42	Eucalyptol (1,8-Cineole)	nd		nd		0.205		nd	
43	Heptanal	nd		nd		0.398		nd	
44	Heptanoic acid	nd		0.690	b	2.833	ab	3.668	a
45	Hexanal	nd		1.145	a	0.712	ab	0.384	b
46	Hexanoic acid	1.816	b	5.077	b	26.161	ab	41.801	a
47	Hexanoic acid, 2-ethyl-	0.473	a	0.158	b	0.192	ab	0.440	a
48	Methyl 2-[4-(1H-indol-2-yl)phenoxy]acetate	nd		nd		nd		1.067	
49	Nonadecane	nd		0.370		nd		nd	
50	Nonanoic acid	0.826	b	nd		1.490	ab	1.346	a
51	Octanoic acid	0.703	c	0.933	bc	4.420	a	4.227	b
52	Oxime-, methoxy-phenyl-_	2.588	a	1.067	b	0.717	b	nd	
53	*p*-Cymenene	nd				0.397	b	0.698	a
54	Pentanoic acid	nd		0.443	b	1.354	b	8.014	a
55	*p*-ethyl-phenol	nd		nd		0.306		nd	
56	Phenol, 2,4-bis(1,1-dimethylethyl)-	nd		nd		nd		2.003	
57	Phenol, 2-ethenyl-, acetate	0.814	a	0.290	a	0.283	a	nd	
58	Phenol, 2-methoxy-	nd		nd		1.645	a	1.153	a
59	Phenylethyl Alcohol	0.722	ab	0.863	a	0.487	ab	0.427	b
60	Propanoic acid, 2-methyl-, 3-hydroxy-2,2,4-trimethylpentyl ester	0.477	a	0.270	ab	0.253	b	nd	
61	Pyrazine, 2,5-dimethyl-	nd		nd		nd		0.386	
62	Silane, ethoxytriethyl-	nd		0.455		nd		nd	
63	Silanediol, dimethyl-	1.360	ab	1.993	a	1.140	b	1.017	b
64	*γ*-Nonalactone	nd		0.340	b	0.488	b	0.771	a

nd—not detected (peak < 0.1% reporting threshold or unreliable integration); mean relative peak areas (%), *n* = 3. Different lowercase letters within the same column indicate statistically significant differences (*p* < 0.05); non-parametric Kruskal–Wallis test was applied.

2,3-Butanediol is responsible for beer aromas with malty and fruity notes [9,37]. In the control wort and in the wort containing 10% hemp malt, the content of this compound was at a similar level (22.257% and 22.261%, respectively). These values were approximately six times higher than in the wort containing 30% hemp malt (3.504%) and eighteen times higher than in the wort with the addition of unmalted hemp seeds (1.206%). The high concentration observed in worts with a lower proportion of hemp suggests that a traditional barley mash promotes the formation of this compound, which is a by-product of yeast metabolism. The drastic decrease in its concentration at higher hemp levels may be associated with changes in the mineral composition of the wort or with the presence of inhibitors affecting yeast metabolic pathways [27]. It is worth emphasising that the results from replicate measurements within the tested worts were characterised by high variability; the standard deviations of 10.1, 4.71, 2.59, and 0.959%, respectively.

The content of 1-butanol-3-methyl, which imparts alcoholic and earthy aromas [38,39], decreased linearly with increasing proportions of malted hemp in the wort. However, the content of this compound in worts produced with barley malt and with the addition of 10% and 30% hemp malt (6.114%, 5.568%, and 4.328%, respectively) did not differ significantly. With the exception of the wort containing 30% hemp malt, these values were significantly higher than those observed in the wort with the addition of unmalted hemp (1.754%). The reduction of 1-butanol-3-methyl in hemp-enriched worts can be regarded as beneficial from a sensory quality perspective.

1-Hexanol, responsible for green, grassy and freshly cut grass notes, is a compound typically formed through lipid oxidation (via the lipoxygenase pathway) present in plant-derived raw materials [40,41]. This compound, which was present at a relatively low level in the control wort (2.509%), showed approximately twofold higher values in worts with the addition of 10% malted hemp seeds and 30% unmalted hemp seeds, and a fourfold increase in the wort containing 30% malted hemp. The marked increase in 1-hexanol concentration in hemp-containing worts directly reflects the introduction of seeds rich in precursors (unsaturated fatty acids), which imparts a characteristic “plant-like” profile.

The concentration of 2-butanone-3-hydroxy was highest in the control wort and lower in the hemp-containing worts. Statistical analysis indicated that differences in content among all malt-based worts were not significant, with the concentration of this compound in the wort containing 30% hemp malt comparable to that in the wort with unmalted hemp seeds. The reduction observed in the wort with unmalted hemp seeds (1.763%) corresponded to only 15% of the concentration in the control wort (11.819%), 17% of that in the wort containing 10% malted hemp (10.049%), and 43% of that in the wort containing 30% malted hemp. Depending on the degree of fermentation, 2-butanone-3-hydroxy accounts for balanced buttery notes, followed by creamy and slightly milky nuances, imparting a milder, sweetish aroma to the wort. 2-Butanone-3-hydroxy is a precursor of diacetyl, which is known for its buttery or toffee-like flavour and imparts a milder, slightly sweet aroma to the wort [33]. The decrease in 2,3-butanediol content following the addition of hemp, particularly unmalted hemp seeds, confirms that the incorporation of hemp is likely to affect yeast metabolism along the pathway responsible for the production of these compounds [33].

Hexanoic acid is characterised by a goaty and rancid odour [34,42]. Its concentration increased exponentially with increasing proportions of hemp in the wort. The content of this acid in the control wort was relatively low, at 1.82%. The addition of 10% and 30% malted hemp to the wort increased the hexanoic acid content to 5.077% (threefold) and 26.161% (fourteenfold), respectively, while the addition of unmalted hemp seeds increased it to 41.801% (twenty-threefold). It should be emphasised that the hexanoic acid content in successive worts was 2.6%, 6.7%, 31.2%, and 46.0%, respectively. This indicates a strong dominance of hexanoic acid aroma in worts containing 30% hemp malt, and an even more pronounced dominance in worts with a 30% proportion of unmalted hemp seeds. The presence of hexanoic acid in beer above the sensory threshold is regarded as a serious sensory defect [34,42]. Such a pronounced increase is likely the result of supplying precursors in the form of lipids from hemp seeds, which leads to an overproduction of fatty acids. The sensory relevance of elevated hexanoic acid levels observed in worts supplemented with unmalted hemp seeds is inferred from reported odor threshold values in the literature rather than direct sensory evaluation [43].

A characteristic spicy profile of beer is determined by 4-vinyl guaiacol, which is responsible for distinctive clove- and spice-like notes sometimes accompanied by slightly smoky nuances, typically found in classic wheat beers. This compound is formed through the decarboxylation of ferulic acid by specialised yeast strains [31]. 4-vinyl guaiacol dominated in the control wort (6.150%), while significant amounts were noted in the wort containing 10% hemp malt (3.983%). Significantly lower concentrations of this compound were found in worts with 30% hemp seed, both malted (1.257%) and unmalted (0.853%). The sensory threshold of this compound is very low (200 ppb), so even small amounts can affect aroma perception. It is worth noting that, although this compound contributes to the characteristic aroma of wheat beers, it is undesirable in lagers and light beers [44].

This decrease suggests that hemp either dilutes ferulic acid precursors or inhibits yeast enzymatic activity, reducing phenolic notes in malt-based worts.

Chromatographic analysis of the worts identified a total of 64 unique volatile organic compounds (VOCs), 43 of which were present at concentrations below 1%. Despite their low levels in the wort, these compounds can significantly impact the final aroma profile of the beer due to their low sensory thresholds. Among this group of compounds with a high aromatic impact, 1-octen-3-ol is noteworthy. Its content tended to be higher only in worts with 10% and 30% malted hemp, 1.223% and 1.517% respectively, compared to 0.678% in the control and 0.672% in the wort with 30% unmalted hemp seed. The presence of this compound in wort may contribute to the preservation of earthy aromas in beer [32].

**Figure 1:**
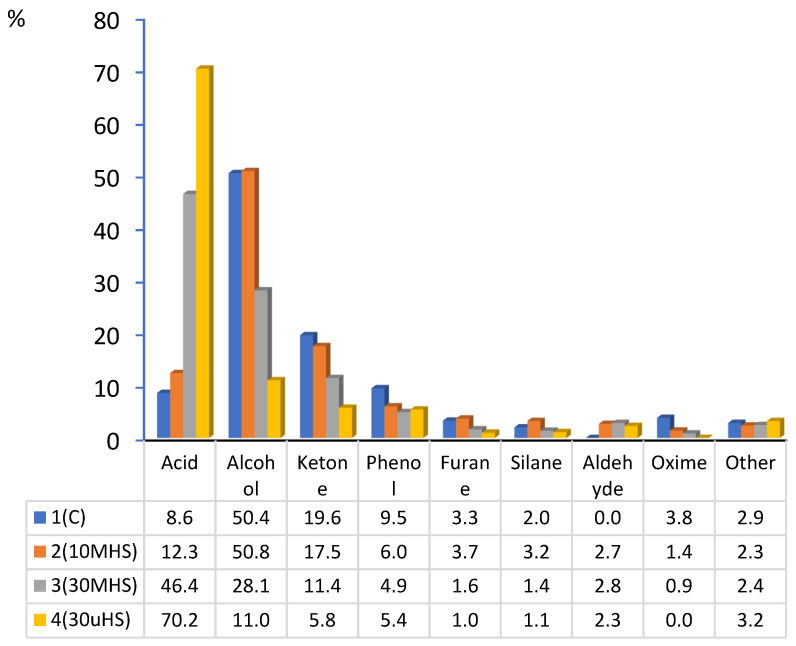
Percentage of chemical compound groups in barley malt worts 1(C), with the addition of 10% hemp malt 2 (10 MHS) and 30% hemp malt 3 (30 MHS) and 30% unmalted hemp seeds 4 (30 uHS).

Another compound with a high aromatic impact, *γ*-nonalaktone, is a lactone that imparts notes of coconut, peach, and fruit. In this research, this compound was found only in worts with a share of hemp with significantly higher content in wort with unmalted hemp seeds (0.771% compared with 0.340% in wort with 10% malted hemp and 0.488% in wort with 30% malted hemp). As a result, this may have an impact on the presence of fruity notes in the final product. Dimethyl trisulfide, which is characterised by an exceptionally low sensory threshold, is present only in worts containing 10% hemp malt, 0.260%. This compound imparts sulfurous, onion-like and marshy aromas, which can noticeably modify the sensory profile even at trace levels. Among the compounds with a medium aromatic impact, benzaldehyde (0.98%) is noteworthy, as it imparts characteristic notes of almond and cherry. In the analysed worts, this compound exhibited an increasing trend with rising hemp content, reaching the highest concentration (1.383%) in wort containing 30% unmalted hemp seeds. This increase may contribute to the sweet, nutty notes of the final product. Another compound, 2-methoxy-4-vinylphenol, was found exclusively in wort containing 30% hemp malt (1.300%); it imparts characteristic clove and spice aromas typical of some wheat beer profiles. Meanwhile, hexanal, found only in hemp worts, is responsible for green, grassy aromas that can enhance the “fresh” character of hemp worts. The highest hexanal concentration was found in worts with 10% hemp malt (1.145%), exceeding the concentration in worts with 30% hemp malt by 1.6 times and the concentration in worts with 30% unmalted hemp seeds by three times. VOCs identified in worts encompass a wide spectrum of chemical classes, such as alcohols, acids, ketones, aldehydes, phenols, furans, silanes, oximes, and others (esters, sulfides, hydrocarbons, pyrazines, lactones, terpenes, alcohols/terpenes, terpenes/ethers) (Figure 1).

From a perspective of chemical groups, the control wort and the wort containing 10% hemp malt were characterised by a predominance of alcohols (over 50%), a moderate ketone content (approximately 18–20%), and a relatively low acid content (7–14%). Such a profile accounts for the classic malty and fruity aromas. The wort with 30% hemp malt exhibits a stronger acid concentration (46.4%), with a simultaneous decrease in alcohol (28.1%) and ketone content (11.4%). This indicates a shift in the aromatic profile towards more acidic, vegetal, and less malty notes. Meanwhile, the wort containing 30% unmalted hemp seed has a clear predominance of acids (70.2%) and a very low alcohol content (11.5%). Such a high acid content gives it an intensely acidic, earthy, and grassy character, distinct from that of barley wort. The relations between the chemical groups described above indicate that increasing the proportion of hemp in the wort leads to an increase in acidity at the expense of alcohol and ketone content. The addition of hemp seed, particularly in malted form and in higher concentrations, significantly changes the aromatic profile of worts, increasing the proportion of VOCs that impart green, herbal, and fatty aromas, and reducing the proportion of substances responsible for traditional phenolic and sweet notes. Variants containing 30% unmalted hemp seed have the most pronounced, vegetal aroma character, while the control wort retains a more balanced and classic aroma profile.

**Figure 2:**
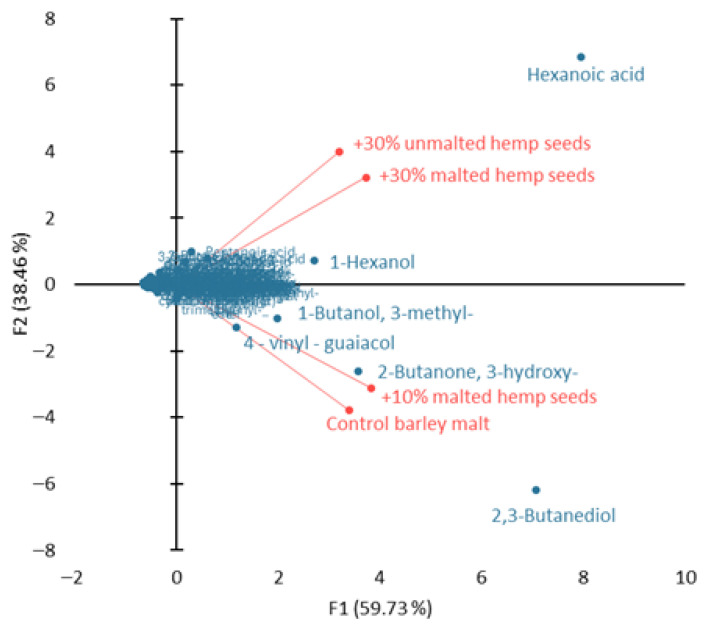
PCA biplot of the volatile organic compounds (VOCs) profile in barley/hemp worts.

The PCA results are presented in Figure 2. The first two principal components explain 98.19% of the total variance. This means that the graph illustrates almost the entire variability in the chemical profile of the worts. The first component (59.73% of the variance) separates the worts primarily according to the share of fatty acids (hexanoic acid) as well as alcohols (1-hexanol and 3-methyl-butanol) and ketones (2,3-butanediol and 2-butanone-3-hydroxy), while the second component (F2 = 38.46%) accounts for the separation associated with the presence of higher alcohols (1-hexanol) and phenols (4-vinyl-guaiacol). The grouping of the control wort and the wort with 10% hemp malt against dominant shares of 2,3-butanediol and 2-butanone-3-hydroxy, which impart creamy and malty aromas, indicates a classic malty and alcoholic wort profile. On the other hand, worts with a high proportion of malted or unmalted hemp seeds constitute a second group of worts with dominant hexanoic acid, which gives these worts intensely acidic and fatty aromatic notes. 1-Hexanol, responsible for green and grassy aromas, and 4-vinyl-guaiacol, responsible for clove-like and spicy aromas, have a significant impact on the separation of worts with the addition of hemp, especially those with the addition of 30% hemp malt and 30% unmalted hemp seeds. The obtained PCA results indicate that the main chemical markers differentiating the worts are: hexanoic acid determining the acidic hemp profile, 2,3-butanediol and 2-butanone-3-hydroxy determining the malt and barley profile, 1-hexanol accounting for green notes, and 4-vinyl-guaiacol which imparts phenolic and spicy aromas. This means that increasing the proportion of hemp in the fermentation process shifts the wort’s aromatic profile from malty and creamy to acidic, vegetal, and phenolic.

When grouped by compound class, the control wort and the wort containing 10% hemp malt were dominated by alcohols (over 50%), with moderate ketone levels (18–20%) and relatively low acid content (7–14%). Such a profile reflects the classic malty and fruity aromas typical of barley beers [9]. Increasing the proportion of hemp led to a marked change in the chemical profile: in wort with 30% hemp malt, acids accounted for 46.4%, while alcohols and ketones dropped to 28.1% and 11.4%, respectively. In wort containing 30% unmalted hemp seeds, acidity reached 70.2%, while alcohol content reached only 11.5%. Increasing the hemp seed content in the wort leads to an increase in acidity at the expense of alcohol and ketone content. This phenomenon is well-documented in the literature, where changes in mash composition, particularly the addition of lipid-rich ingredients, lead to a shift in yeast metabolism toward the production of fatty acids and “green” profile compounds (e.g., hexanol), while simultaneously limiting the synthesis of esters and higher alcohols [40,45]. The addition of hemp seeds, especially in unmalted form and in higher concentrations, significantly changes the aromatic profile of worts, increasing the share of VOCs that give green, herbal and fatty aromas, and reducing the share of compounds responsible for traditional phenolic and sweet notes.

The occurrence of selected VOCs exclusively at 10% hemp addition suggests a nonlinear, matrix-dependent aroma formation mechanism, likely driven by competitive enzymatic pathways and matrix effects during mashing leading to metabolic redirection, rather than analytical inconsistency [39].

Overall, high hemp addition significantly modifies yeast metabolism and the chemical balance of wort. Moderate additions (about 10%) maintain malty and fruity notes while introducing subtle herbal aromas, suitable for novel craft-style beers.

### 3.2. Mineral Composition of the Wort

The mineral composition of wort is a key factor influencing yeast physiology, fermentation, and, consequently, the sensory profile of the final product [27]. Table 2 presents basic statistics for eleven elements across four wort variants, along with an assessment of the significance of differences in pairwise comparisons. The key macronutrients differentiating the worts were potassium, magnesium, phosphorus, and calcium, while the micronutrients included manganese, iron, and sodium.

The control wort produced from barley malt contained 5070 mg/kg of potassium, while all worts with added hemp showed a drastic reduction to 286–363 mg/L (a decrease of over 90%). Such a significant reduction in potassium content could have had a significant impact on yeast activity during fermentation and could have partially determined the observed changes in the VOC profile. Potassium is the most important intracellular cation in yeast, crucial for maintaining membrane potential, regulating pH, and activating numerous enzymes [46]. Such a significant decrease in potassium content could have substantially affected yeast activity, inducing osmotic and ionic stress. Potassium deficiency in worts with the addition of malted and unmalted hemp seeds may lead to disruptions in the production of higher alcohols and contribute to an increased production of fatty acids [26], which is related to the reported increase in hexanoic acid from 1.8% to 41.8% [27].

Magnesium showed an opposite upward trend, rising from 122 mg/kg in the control to 139–163 mg/kg in hemp-added worts, with the highest value in the wort with unmalted hemp (163 mg/kg). As a cofactor for over 600 enzymes, including those involved in glycolysis and ATP synthesis, this increase could partially compensate for the low potassium content [21,47].

Phosphorus also rose progressively with hemp addition (219 → 429 mg/kg), supporting energy metabolism, nucleic acid synthesis, and cell membrane formation [48].

Calcium peaked in the 10% hemp malt wort (26.4 mg/kg, +36% vs. control) and stabilised at higher hemp additions. Calcium supports yeast flocculation, although excess may cause oxalate precipitation [27].

Manganese content increased with increasing hemp addition, rising from 0.69 mg/kg in the control wort to 0.96 mg/kg in worts containing 30% hemp (a 40% increase). Manganese serves as an enzyme cofactor, protects cells from oxidative stress, and may affect the progression of biochemical processes during fermentation [49,50].

Zinc, with a content of 0.42–0.70 mg/kg, which is below the WHO limit (5 mg/L), is a key trace element involved in protein synthesis and the structural stability of alcohol dehydrogenase, thereby supporting healthy yeast metabolism [51,52].

Iron, at concentrations ranging from 29.5 to 31.9 mg/kg, did not differentiate the worts tested. Iron is essential in *Saccharomyces cerevisiae* yeasts as a redox-active cofactor for numerous metabolic processes, including mitochondrial respiration, haem and iron–sulfur cluster formation, lipid and sterol biosynthesis, and DNA and RNA synthesis. Iron deficiency impairs these pathways, leading to altered metabolism and reduced cellular function [53]. Conversely, excess iron can catalyse redox reactions that generate reactive oxygen species (ROS), which damage cellular components (e.g., rRNA cleavage and oxidative stress), impair growth or viability, and disrupt fermentation performance [54].

In turn, sodium with the addition of hemp seeds showed a decrease. Sodium plays a key role in yeast by contributing to osmotic balance, maintaining cellular turgor, and supporting the function of membrane transporters involved in nutrient uptake and ion homeostasis. In the wort, the observed decrease in sodium concentration from 12.4 mg/kg in the control to 6.6 mg/kg in the wort with unmalted hemp seeds (a 47% reduction) may reduce yeast viability or stress tolerance during fermentation. Consequently, this reduction could affect the fermentation performance, potentially slowing sugar metabolism and altering the production of ethanol and secondary metabolites in the beer [55,56].

Regardless of the wort, cadmium and lead were recorded in marginal amounts, below the limit for beverages of 0.10 mg/L, but in amounts higher than for water (0.01 mg/L). Zinc content, insignificantly different between worts, in the range of 0.42–0.70 mg/kg, was clearly below the WHO limit of 5 mg/L for beer and supported yeast metabolism. Similarly, aluminium content, associated with beer cloudiness, can be considered sensorially safe, despite significant variation between worts, with the highest content in the control wort (6509 mg/kg), significantly lower levels in worts with malted hemp seeds (3594 mg/kg and 3300 mg/kg), and the lowest found in the wort with unmalted hemp seeds (2943 mg/kg). However, the phytoremediation capacity of hemp presents a dual concern: while its efficient uptake of minerals from the soil can enhance its nutritional profile, it also raises safety considerations, as the plant is capable of accumulating heavy metals when cultivated in contaminated environments [57].

In summary, hemp addition drastically reduced potassium while increasing magnesium, phosphorus, calcium, and manganese, creating a mineral profile that may both challenge yeast activity and support fermentation, with safe levels of trace metals ensuring favourable sensory conditions.

### 3.3. Study Limitations

This study focused on wort-level analyses under controlled laboratory conditions and did not include full physicochemical characterisation of hemp seeds or sensory evaluation of the final product. Aroma-related interpretations are therefore based on chemical profiling rather than direct sensory assessment. These limitations are a direct consequence of the stepwise experimental design applied across the research series and will be addressed in subsequent studies involving finished beer.

## 4. Conclusions

The results of the mineral composition analysis in the worts indicate that the malting process of hemp seeds can partially limit the release of elements crucial for fermentation, resulting in less pronounced changes in the aromatic profile of worts containing malted seeds compared to those with unmalted seeds. The addition of hemp seeds, particularly in unmalted form or at higher proportions, significantly modifies both the mineral content and the aromatic profile, primarily through a substantial decrease in potassium, along with changes in magnesium, phosphorus, calcium, and manganese concentrations, which affect yeast activity and fermentation dynamics, leading to alterations in the volatile organic compound (VOC) profile.

Consequently, increasing the proportion of hemp seeds shifts the wort aroma towards acidic, green, herbal, and fatty notes, while attenuating classical malt and phenolic accents, whereas the control wort maintains a more balanced and traditional sensory profile. Moreover, the limited mineral release during malting may explain the milder aromatic changes observed in worts with malted hemp seeds, suggesting that the observed sensory differences have a biochemical basis linked to the availability of key minerals.

The addition of hemp seeds to the mash significantly and dose-dependently alters the composition of aromatic compounds in the beer wort, shifting the profile from a classical malty/fruity character to an acidic, plant-like one.

Key chemical markers of this change include a decrease in 2,3-butanediol and 4-vinylguaiacol, alongside a substantial increase in hexanoic acid and 1-hexanol. The rise in hexanoic acid to levels that dominate the VOC profile poses a significant sensory challenge.

The form in which hemp seeds are added is critical: unmalted seeds induce more pronounced changes in both the aromatic and mineral profiles than malted seeds, suggesting that the malting process partially mitigates negative effects.

The primary cause of changes in the VOC profile is most likely the significant alteration of wort mineral composition, particularly the critical deficiency of potassium, which induces metabolic stress in yeast and redirects their biochemical pathways.

The application of 30% hemp seeds, particularly in unmalted form, generates an aromatic profile that may be sensorially unacceptable in the finished beer due to the dominance of rancid notes. Future studies should focus on lower hemp addition levels and on optimising hemp malting to enhance mineral bioavailability.

## Figures and Tables

**Table 2:** Mineral compounds in worts with the addition of malted and unmalted hemp seeds (mg/kg).

Mineral Compound	Statistics	Control Barley Malt		+10% Malted Hemp Seeds		+30% Malted Hemp Seeds		+30% Unmalted Hemp Seeds	
Al	Mean	6.509	a	3.594	b	3.300	b	2.943	c
	SD	0.218		0.345		0.166		0.228	
Ca	Mean	19.327	b	26.390	a	21.674	b	23.594	ab
	SD	0.055		4.724		1.521		1.136	
Cd	Mean	0.010	a	0.005	a	0.013	a	0.008	a
	SD	0.006		0.005		0.008		0.008	
Fe	Mean	31.516	a	29.844	a	31.875	a	29.548	a
	SD	0.023		0.264		4.930		0.617	
K	Mean	5070.085	a	329.801	b	362.639	b	286.019	b
	SD	1252.413		54.652		56.978		164.555	
Mg	Mean	121.789	c	143.526	b	138.656	b	162.857	a
	SD	0.316		7.080		1.743		0.474	
Mn	Mean	0.689	c	0.857	b	0.960	a	0.964	a
	SD	0.003		0.067		0.011		0.017	
Na	Mean	12.389	a	8.150	b	7.024	bc	6.614	c
	SD	0.101		1.776		0.734		1.102	
P	Mean	219.365	d	288.523	c	344.377	b	429.139	a
	SD	0.958		6.842		10.162		11.330	
Pb	Mean	0.038	a	0.035	a	0.050	a	0.077	a
	SD	0.046		0.021		0.042		0.038	
Zn	Mean	0.425	a	0.699	a	0.642	a	0.624	a
	SD	0.007		0.177		0.175		0.097	

SD—standard deviation, (n = 3). a, b, c, d the same letters point out for insignificant differences according to Tukey’s test at *p* < 0.005.

## Data Availability

All relevant data are contained within the article.
